# Easy and Effective Method for α-CD:N_2_O Host–Guest Complex Formation

**DOI:** 10.3390/ijms25105472

**Published:** 2024-05-17

**Authors:** Tsveta P. Sarafska, Maya I. Spassova, Todor M. Dudev, Stiliana M. Pereva, Simeon D. Stoyanov, Tony G. Spassov

**Affiliations:** 1 Faculty of Chemistry and Pharmacy, Sofia University “St. Kl. Ohridski”, 1164 Sofia, Bulgaria; nhtth@chem.uni-sofia.bg (T.P.S.); nhtms@chem.uni-sofia.bg (M.I.S.); ohtttd@chem.uni-sofia.bg (T.M.D.); nhtsp@chem.uni-sofia.bg (S.M.P.); 2 Singapore Institute of Technology, 10 Dover Drive, Singapore 138683, Singapore; simeon.stoyanov@singaporetech.edu.sg

**Keywords:** alpha cyclodextrin, nitrous oxide, inclusion complex, stability

## Abstract

α-CD:N_2_O “host-guest” type complexes were formed by a simple solid–gas reaction (N_2_O sorption into α-CD) under different gas pressures and temperatures. The new N_2_O inclusion method applied in the present study was compared with the already known technique based on the crystallization of clathrates from a water solution of α-CD saturated with N_2_O. A maximum storage capacity of 4.5 wt.% N_2_O was achieved when charging the cyclodextrin from a gas phase. The amount of included gas decreases to 1.3 wt.% when the complex is stored in air at 1 atm and room temperature, analogous to that achieved by the crystallization of α-CD:N_2_O. Furthermore, it was shown that the external coordination of N_2_O to either the upper or lower rim of α-CD without hydration water displacement is the preferred mode of binding, due to hydrogen bonds with neighboring -OH groups from the host macrocycle and three of the hydration water molecules nearby. The capacity of α-CD to store N_2_O and the thermal stability of the α-CD:N_2_O complex demonstrated promising applications of these types of complexes in food and beverages.

## 1. Introduction

Cyclodextrins (CDs) are one of the most widely used families of host molecules for including different small (N_2_, CO_2_, NH_3_, N_2_O) and larger (ibuprofen, naproxen, ketoprofen, miconazole, etc.) guest molecules. Thanks to their lipophilic inner cavity and hydrophilic outer surface, α-, β- and γ-CDs are suitable cavitands for the formation of inclusion complexes. These “host-guest” complexes are objects of growing interest due to their application in pharmaceutics [1,2,3,4,5,6,7,8,9,10,11,12,13,14,15,16], cosmetics [17,18,19,20,21], nutrition [22,23,24], gas storage, encapsulation and transport [22,24,25,26,27,28,29,30], biosensing and the biomedical field [1,31,32].

In the food industry, CDs are used for the stabilization of flavors, food additives and vitamins, the protection of food components against oxidation, the elimination or reduction of undesired tastes and the improved shelf life of food products [22,33]. In pharmacy, CDs are applied for forming inclusion complexes resulting in increased drug solubility [10,11], in cosmetics, CDs are applied for capturing odors [17,18,19,20,21], etc. The increased use of CDs in these industries is also due to their nontoxicity and relatively low price.

Cyclodextrins and cyclodextrin-based nanosponges (CD-NSs) are efficiently used as gas carriers as well [22,24,25,26,27,28,29,30]. Three different gases—oxygen, carbon dioxide and 1-methylcyclopropene—have mainly been applied [22]. They managed to entrap a large amount of CO_2_ in β-CD carbonate nanosponges at atmospheric pressure and room temperature and as a result of the strong interaction between the guest–host molecules, a significant amount of CO_2_ was retained under vacuum at 373 K. These nanosponges were also suitable for oxygen encapsulation and were capable of releasing it for a long period [22]. Recently, an in-depth study on improving the gas encapsulation capacity in cyclodextrins was published [24]. The formation of longer channels in the cyclodextrin molecules, in which gas molecules are trapped during encapsulation, was proposed to increase the gas encapsulation capacity. Another suggested approach to increase the encapsulation capacity was to modify the structure of CD molecules so that they can occupy more gas in their cavities [24].

Among the cyclodextrins, α-CD is characterized with the smallest internal cavity and is proven to be a proper host for including/storing of mainly small gas molecules like CO_2_, N_2_ and N_2_O [28,34,35]. A patent from Kraft Foods Inc. (USA, Chicago, Illinois) was focused on the production of solid–gas clathrates based on cyclodextrins [34]. The production of cyclodextrin-based clathrates of only α-CD with N_2_O and CO_2_, applying from 2 to 30 atm of gas pressure to introduce the gas in the water solution of the CDs, was reported [34]. In our previous study [28], N_2_O has been included into α-CD, applying 10–30 atm of N_2_O gas pressure to dissolve the gas in the water solution of the α-CD. The α-CD:N_2_O complexes that formed precipitated from the solution. The advantages of N_2_O compared to CO_2_ were used in conventional backing powders, as foam boosters are connected with some practical benefits. As is well known, CO_2_ is released due to the chemical reaction between carbonate salt (Na or K) and acid, which leads to an unpleasant taste (CO_2_ is fizzy and salt (KCl or NaCl) is produced). Moreover, CO_2_ is too soluble, and bubbles grow quickly.

Besides our work [28], the patent of Nestle/Kraft/Kievit [34] also presented results on the formation of foam boosters based on the clathrates of dextrins and some gases (N_2_, N_2_O, CO_2_). Furthermore, in addition to the important practical question of whether N_2_O can be stored in α-CD and in what quantity, there are some other fundamental questions worth answering, as follows: (i) does the inclusion of N_2_O into the α-CD result in the displacement of internal water molecules? (ii) do H_2_O and N_2_O molecules compete in the process of inclusion into α-CD? (iii) does the stability of the α-CD:N_2_O complex depend on how it is formed (by gas absorption or co-crystallization)? Therefore, in the present work combining experimental and theoretical methods, we investigated for the first time systematically the inclusion of N_2_O into α-CD from an N_2_O gas phase. In addition to the capacity of α-CD to store nitrous oxide, the competition of N_2_O and H_2_O molecules to occupy the α-CD cavity was also studied.

## 2. Results 

### Theoretical Modeling

**Figure 1:**
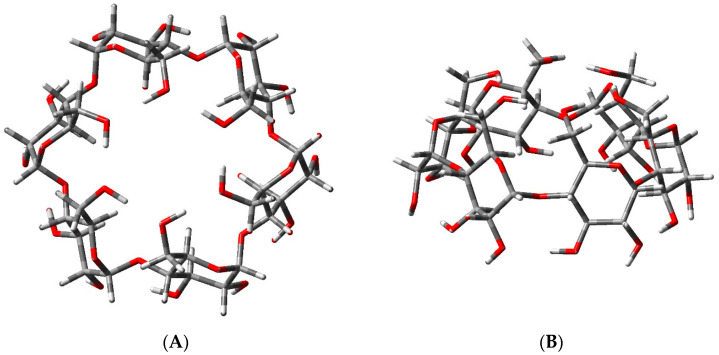
M062X/6-31G* fully optimized structure of α-cyclodextrin: (**A**) top view and (**B**) side view.

**Figure 2:**
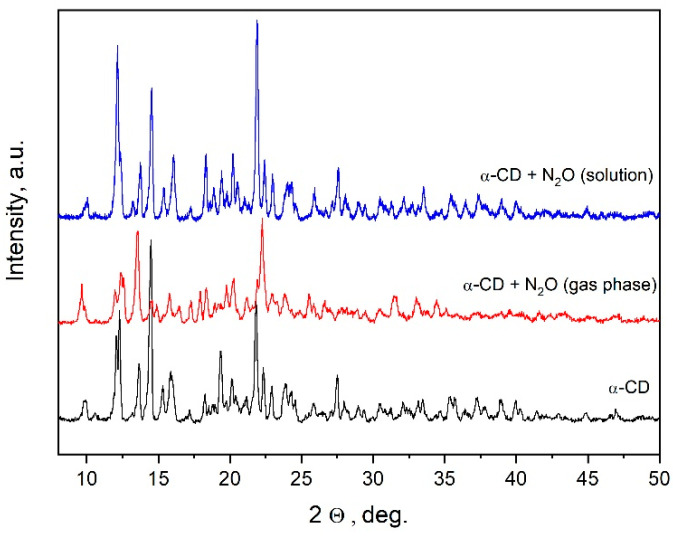
X-ray diffraction (Cu-Ka) of pure α-CD and of the complexes formed by the two different methods.

α-CD and its complexes with water and N_2_O were modeled explicitly. The most energetically favorable structure of the α-CD cavitand was taken into account, i.e., the “closed” configuration featuring internal hydrogen bonds between the hydroxyl groups decorating the macrocycle’s upper and lower rims, arranged in a “head-tail” fashion [36] (Figure 1). The host macrocycle was modeled in its fully hydrated form (α-CD:6wat; Figure 2) as both experiment and theory have shown that the central cavity may accommodate up to six water molecules at an ambient temperature [36].

Density functional theory (DFT) calculations were performed. The geometries of all participating entities were optimized at the M062X/6-31G* level of theory using the Gaussian 09 package of programs [37]. The Minnesota M062X functional in combination with a split-valence double-ζ basis set was employed in the calculations as it has been proven to be dependable in reproducing the geometrical parameters of a number of macrocyclic cavitands and their inclusion complexes [36,38,39,40]. Vibrational frequencies (none of them imaginary) and thermal energies, including zero-point energies, E_th_, at 25 °C and 1 atm pressure were evaluated at the same level of theory. The electronic energies, E_el_, were corrected at a higher theoretical level (M062X/6-31+G**) via single-point calculations performed on the M062X/6-31G* optimized structures. 

Two scenarios for the N_2_O binding to the host macrocycle were considered:

- The external coordination of the guest molecule to either the upper or lower rims of α-CD without dislodging hydration water molecule(s).
  [α-CD:6wat] + N_2_O → {[α-CD:6wat]N_2_O} (1)
- N_2_O binding inside the central cavern of the cavitand accompanied by a water molecule displacement.
  [α-CD:6wat] + N_2_O → [α-CD:5wat:N_2_O] + 1wat (2)
  Taking into consideration the differences in the respective quantities between the products and reactants in the above equations, the enthalpy of the process, ΔH, was evaluated as
  ΔH = ΔE_el_ + ΔE_th_ + ΔpV (3)
  where ΔpV is a work term accounting for the difference in the number of moles in the two sides of Equations (1) and (2); it is −0.59 kcal/mol for Equation (1) and 0 kcal/mol for Equation (2).

## 3. Discussion

In the present work, a promising synthetic approach has been applied for α-CD:N_2_O complex formation based on the direct N_2_O loading of dry cyclodextrins from an N_2_O gas phase. This approach is an alternative of the “cyclodextrin-gas” type clathrates’ formation by crystallization from a water solution of CDs saturated with N_2_O [28], and reduces some N_2_O transport difficulties. The X-ray diffractograms of the complexes formed by the two approaches are compared in Figure 2. The X-ray diffractogram of the α-CD studied shows that it has a well-defined crystal structure, with a complete coincidence of the diffraction peaks with those characteristic of the cage-type crystal structure of the cyclodextrin [41,42]. From the comparison with the diffractograms of the α-CD:N_2_O complexes, it can be seen that the crystal structure of α-CD does not change significantly due to the incorporation of N_2_O. However, some microstructural difference was observed between the complexes formed by the two methods. These differences are mostly visible in the altered intensities of the diffraction peaks and also in the disappearance of some peaks in the method of loading cyclodextrin directly from a gas phase. The solution-derived complexes hardly change the structure of pure α-CD; both the position of the peaks and, in general, their intensities are preserved and correspond to alpha-Cyclodextrin hexahydrate [43]. However, the gas-phase-derived complexes show a change in some interplanar distances, a difference which can be attributed to both the different amount of N_2_O incorporated in the cyclodextrin and the applied pressure required for its incorporation. Thus, the X-ray patterns can be considered as a sort of fingerprint of the complexes formed by the two methodologies, which further in the text will be denoted as α-CD:N_2_O (solution) and α-CD:N_2_O (gas phase).

**Figure 3:**
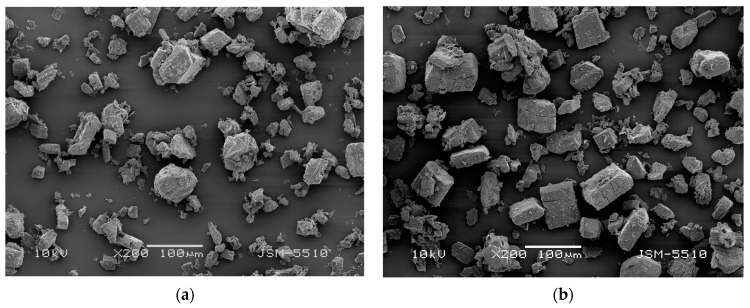
SEM micrographs of α-CD (**a**) and α-CD:N_2_O complex (**b**).

SEM images of the initial α-CD and of the α-CD:N_2_O complex, presented in Figure 3, show that the initial morphology and size of the particles are not changed due to the gas absorption into the cyclodextrin. However, a comparison of the micrographs reveals distinct cracks on the larger particles of the complex, apparently related to a cracking process induced by the gas absorption in them. At the same time, particle decrepitation is practically not observed.

**Figure 4:**
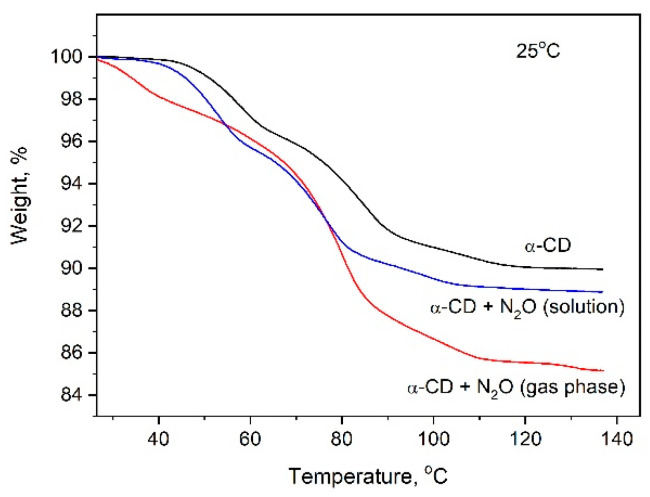
TG curves of α-CD:N_2_O complexes formed by the two different methods (TG curve of pure α-CD is also presented for comparison).

**Figure 5:**
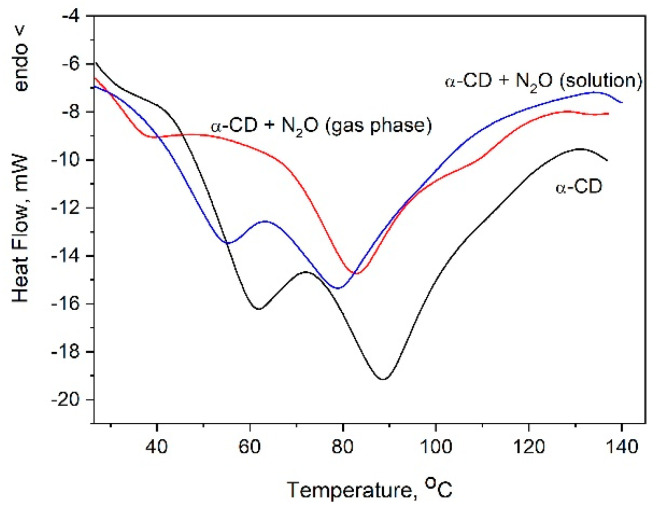
DTA analysis of α-CD:N_2_O complexes formed by the two different methods. DTA curve of pure α-CD is also presented.

To determine the amount of N_2_O included into the cyclodextrin by the new methodology and compare it with that achieved through the crystallization of α-CD:N_2_O from a water solution saturated with nitrous gas, thermogravimetric (TG) analysis has been applied. The TG curves of pure α-CD and of the complexes obtained by different methods are presented in Figure 4. The observed difference is significant and is reflected in both the amount of included gas and its thermal stability. The loading of the α-CD with N_2_O from the gas phase leads to a much higher amount of desorbed N_2_O, and its release upon heating occurs mainly at about 80–110 °C, evidenced by the mostly extended second and third TG steps. This result was confirmed also from the DTA analysis, Figure 5. Another distinct difference between the thermal behavior of the two complexes is expressed by the first low-temperature TG step in the gas-phase-derived complex, absent in its counterpart, which quantitatively corresponds to the release of 1–2 molecules of water (for 1 molecule α-CD).

Here, it should also be noted that our recent study demonstrated the insertion of N_2_O into the α-CD cavity without displacing the internal water molecules [44], as the latter even have a stabilizing role due to preferential interactions between the water and nitrous molecules in the cyclodextrin cavity rather than with the α-CD walls. This result is also a reason to determine the amount of stored nitrous oxide by the difference in the weight reduction of pure α-CD and that of the α-CD:N_2_O complex during annealing.

**Figure 6:**
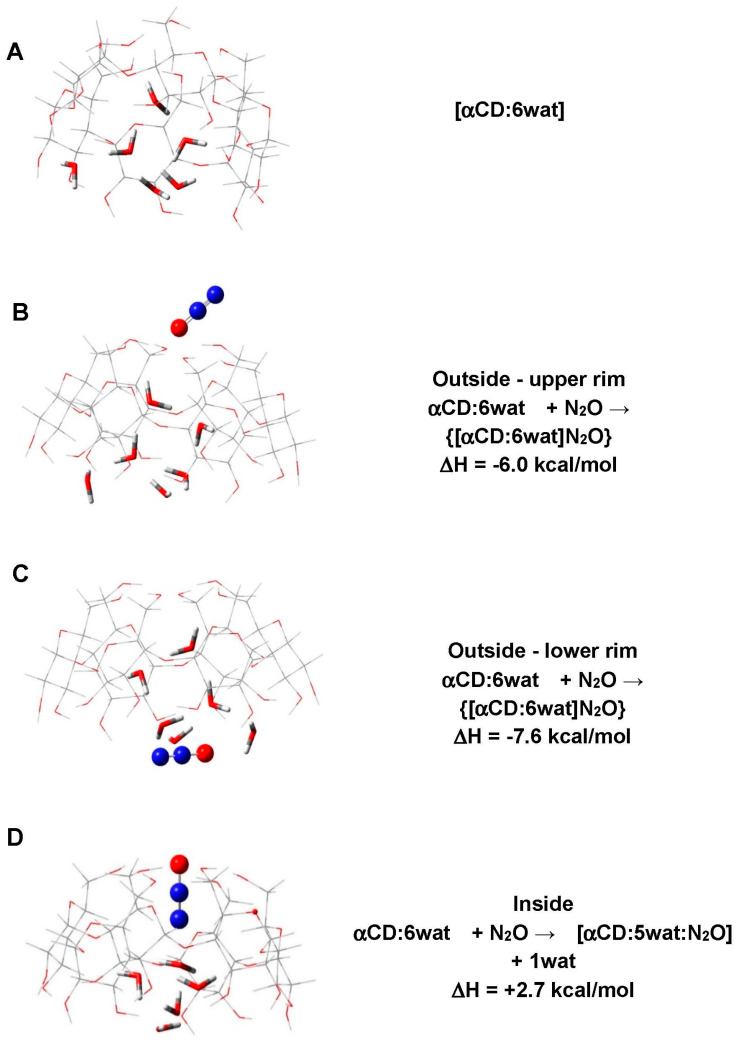
M062X/6-31G* fully optimized structures of (**A**) [α-CD:6wat] complex, (**B**) {[α-CD:6wat]N_2_O} complex with N_2_O bound to the upper rim of the host, (**C**) {[α-CD:6wat]N_2_O} complex with N_2_O bound to the lower rim of the host, and (**D**) [α-CD:5wat:N_2_O] complex, and the respective formation enthalpies (in kcal/mol).

DFT calculations shed further light on the intimate mechanism of N_2_O binding to the host macrocycle. The results are represented in Figure 6. As seen, the external coordination of N_2_O to either the upper or lower rim of α-CD without hydration water displacement is the preferred mode of binding evidenced by negative ΔHs (Figure 6B,C). The calculations suggest that the guest’s association with the lower rim of α-CD (ΔH = −7.6 kcal/mol; Figure 6C) is more advantageous than that with the upper rim (ΔH = −6.0 kcal/mol Figure 6B). The alternative process—the binding of N_2_O inside the internal cavity concomitant with a water molecule release—is not favorable (positive ΔH in Figure 6D). This may be due to the intrinsic stability of the hexa-water cluster residing in the host’s cavern whose disruption, by the attacking gas, destabilizes the resultant complex (Figure 6D).

**Figure 7:**
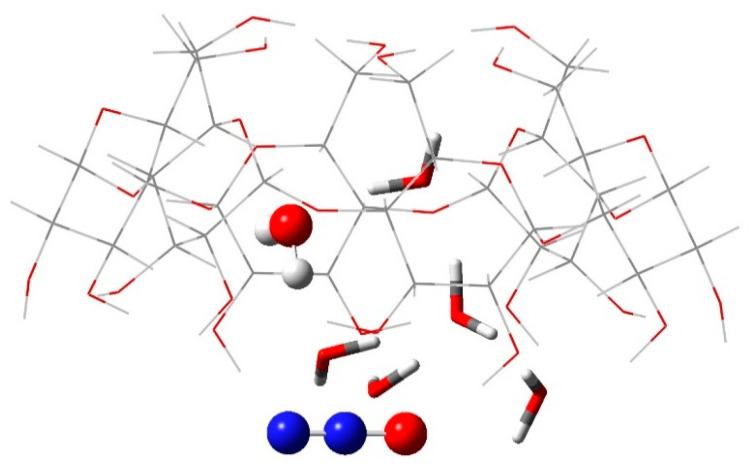
M062X/6-31G* fully optimized structure of {[α-CD:6wat]N_2_O} complex with N_2_O bound to the lower rim of the host. The most weakly bound water molecule inside the macrocycle cavity is shown in a ball-and-stock representation.

Importantly, the computations allow for a detailed assessment of the energy profile of hydration water molecules upon N_2_O binding. Operating on the structure from Figure 6C, the calculations reveal that the bound N_2_O forms hydrogen bonds with neighboring -OH groups from the host macrocycle and three of the hydration water molecules nearby. Thus, the latter are additionally stabilized by 1 to 4 kcal/mol as compared to the uncomplexed [α-CD:6wat] construct. However, the shift/flow of electron density toward the N_2_O binding site leads to the decreasing/depleting of the electron density in the other, more distant part of the water cluster, resulting in the weakening of the respective electrostatic interactions and rendering the other three water molecules somewhat destabilized. The one suffering the highest degree of destabilization (by 1 kcal/mol) is shown in Figure 7. This result agrees well with the observed release of 1–2 water molecules at a significantly lower temperature in the case of the gas-phase-derived complex (see Figure 4).

DTA analysis also confirms the results of the TG measurements and shows once again the influence of the included N_2_O on the thermal behavior of the cyclodextrin, Figure 5. The observed low-temperature thermal peak for the complex formed from the gas phase is absent in the pure cyclodextrin and in the complex formed by crystallization. This fact together with the result of the TG analysis confirm the finding based on the quantum calculations that when the oxide attacks the α-CD, one of the internal water molecules moves to a less favorable position and is released at lower temperatures.

**Figure 8:**
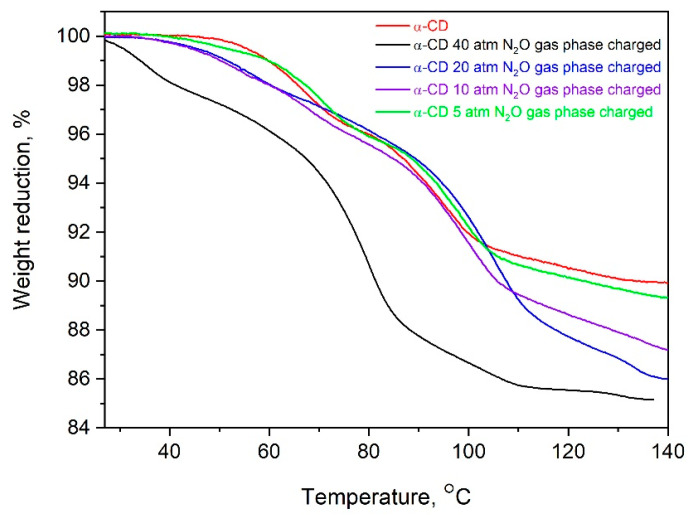
TG curves of α-CD:N_2_O complexes formed by N_2_O absorption at different pressures and constant room temperature.

The amount of N_2_O inclusions in α-CD was found to depend on the applied pressure. Figure 8 shows this dependence, revealing a faster increase of up to 20 atm, after which the pressure dependence weakens. An important result is that the maximum determined amount of absorbed N_2_O corresponds to a molar ratio of α-CD:N_2_O = 1:1. This suggests that one N_2_O molecule is bound to one α-CD molecule, and as mentioned above, without displacing the internal cyclodextrin waters. The real amount can reach 4–4.5 wt.% (more than 20 mL gas per 1 g clathrate) if one takes the final difference in the weight decrease of the clathrates compared to that of the pure α-CD.

**Figure 9:**
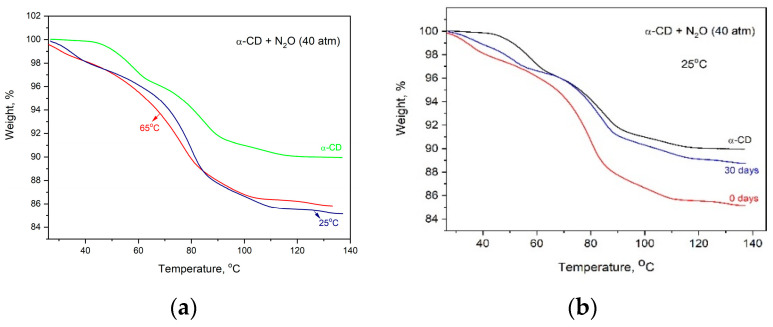
TG analysis of the α-CD:N_2_O complexes formed at two different temperatures at a pressure of 40 bar (**a**) and of the complexes formed at 40 atm pressure and then stored for different times in air at room temperature (**b**).

Attempts were also made to vary the temperature at which N_2_O was loaded into the α-CD by holding the pressure constant (40 atm), as shown in Figure 9. This analysis aimed to accelerate the diffusion of N_2_O into the cyclodextrin crystalline particles. The largest amount of incorporated gas was observed at 25 °C, and at 65 °C, it was nonsignificantly less, thus proving on the one hand that at this pressure, the N_2_O inclusion in the cyclodextrin is thermodynamically favored at both temperatures, and on the other hand, that even at 25 °C, the gas succeeds to enter the core of the α-CD particles.

To prove the practical significance of this method of α-CD:N_2_O complex formation, its stability at room temperature was determined as well. Figure 9b shows the TG curves of the complexes formed at 40 atm pressure after resting for 30 days in air at room temperature (25 °C).

**Figure 10:**
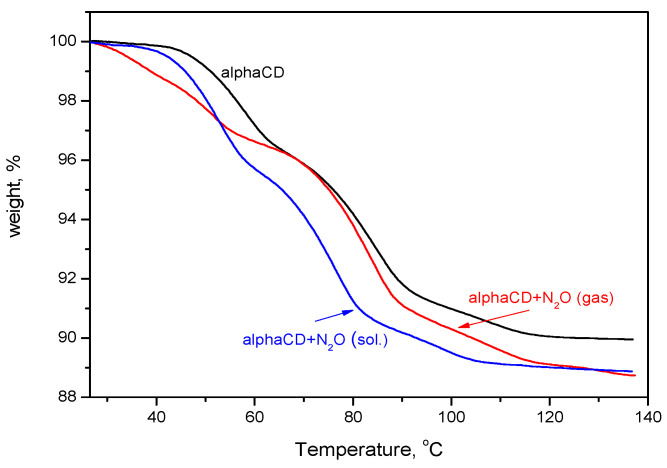
TG curves of α-CD:N_2_O complexes formed by the two different methods stored at ambient conditions for 1 month.

It can be seen from the figures that after 30 days of storage, the amount of included N_2_O decreases to about 1.2–1.3 wt.%, independent of the temperature at which the gas was absorbed into the cyclodextrin, and this value is maintained for a long time. It is also important to stress that this value is close to the amount of N_2_O stored by its inclusion into α-CD from a solution saturated with N_2_O, as seen in Figure 10. There is, however, some difference in the thermal behavior of the incorporated gas, although the final decrease in the sample weight of both clathrates is the same. While the N_2_O introduced into the α-CD through the solution is released up to about 80 °C, as evident from the elongated first two TG steps (in comparison with the TG curve of the pure α-CD), the N_2_O included from a gas phase leaves the cyclodextrin in the range of 80–120 °C, visible from the extended high temperature TG steps, as shown in Figure 10. Generally, the TG curve of α-CD:N_2_O (solution) contains the same steps as the pure α-CD and does not differ substantially from the TG of the “fresh” as-crystallized α-CD:N_2_O (from a solution). In both cases (as-crystallized α-CD:N_2_O clathrate and after 30 days at 25 °C), the start of the noticeable sample weight decrease is ahead of that of the pure α-CD with about 5–10 °C. The TG curves of the complex obtained from a gas phase, however, contain a clearly distinguished low-temperature step in the temperature range of 30–65 °C. The height of the low-temperature step does not change significantly with the time of sample storing at 25 °C. After 30 days, the TG curve still contains the low-temperature weight decrease and its height is about 1–1.2 wt.%, as seen in Figure 10. Since we have shown by theoretical modeling that when N_2_O binds to the α-CD rims, one of the water molecules is energetically destabilized, the preservation of the low-temperature TG step, even when the sample is left in air at RT, proves once again the stability of the α-CD:N_2_O complex.

**Figure 11:**
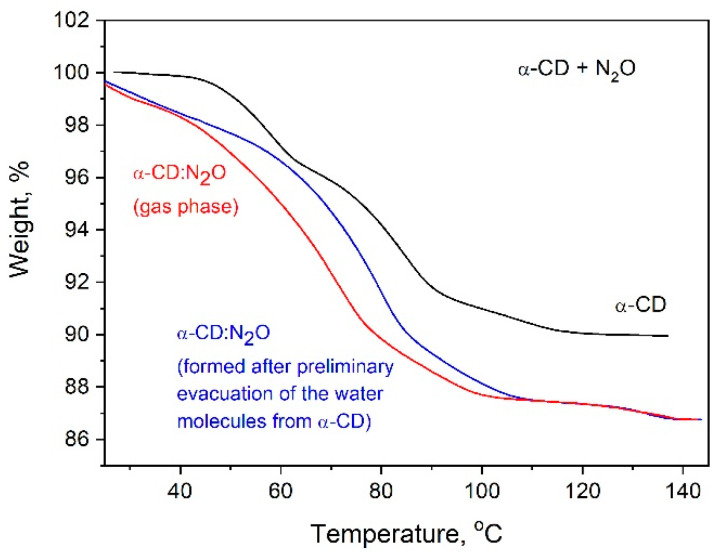
TG curves of α-CD:N_2_O complexes obtained by gas phase N_2_O loading of source α-CD with the internal water and α-CD after removal of the internal waters.

Attempts were also made to initially remove the internal water molecules of α-CD and then to fill its cavern with pressurized N_2_O. For this purpose, α-CD samples were heated to 100 °C for 1 h under vacuum to release the water, and then the samples were charged with N_2_O under a pressure of 40 bar. Although the water was separated from the cyclodextrin and the empty cavity was then filled with N_2_O only, when the samples were exposed to air, they reabsorbed the required amount of water (about six molecules per molecule of α-CD), which can be clearly seen in Figure 11. The results shown in Figure 11 also indicate that in this case, a comparable loading capacity of the α-CD with N_2_O is achieved, accompanied by a rapid recovery of the internal cyclodextrin waters when the complex is vented. This result proves that even with an empty cavity, N_2_O molecules attach to one of the two α-CD rings, leaving a possible pathway for the water molecules to enter the cavity when the complex was exposed to air.

## 4. Materials and Methods

Using dry α-CD powder (as received from the producer Wacker Chemical Corp. Adrian, MI, USA) and N_2_O (purity 99.99%) under pressure, host-guest α-CD:N_2_O complexes were synthesized. Thick-walled stainless-steel reactors were specially designed for this purpose and fitted with appropriate gas inlet valves. For the N_2_O loading of α-CD, the pressure was varied in the range of 5–40 atm and the temperature ranged from 25 to 85 °C.

DTA/TG (TA SDT 650) methods were used to characterize the thermal behavior of the complexes and the amount of stored gas. The microstructural characteristics of the host-guest complexes were determined using X-ray diffraction (Bruker D8 Advance diffractometer with Cu-Kα radiation, Karlsruhe (Germany)).

## 5. Conclusions

It has been shown that it is possible to include noticeable amounts of N_2_O into α-CD in an affordable and efficient manner by a simple solid–gas reaction (N_2_O sorption into α-CD). The N_2_O gas pressure required to achieve maximum capacity at temperatures from 25 to 85 °C is 40 atm. A key result of the study is also the fact that when N_2_O attaches to the α-CD molecule, the available internal water does not leave its cavity, but on the contrary, plays a positive role with respect to the stability of the included gas. Furthermore, in full agreement with the present findings, our recent calculations suggested that the richer the water cloud around the guest entity and the more elaborate the resultant hydrogen bond network in the cavity, the more efficient the encapsulation process is [40]. The applied approach, based on a gas (N_2_O)—solid (α-CD) reaction, is an alternative of the cyclodextrin-gas type clathrates’ formation by crystallization from a water solution of CDs saturated with N_2_O. Both the amount of gas stored by the two methodologies and its stability at room temperature were compared and it was found that the new method allows the initial inclusion of a much larger amount of gas into the cyclodextrin (about 4.5 wt.%) compared to the crystallization method. When stored in air at 1 atm and room temperature, this amount decreases to a stable value of 1.3 wt.%. This value persists for a long time and is equal to that achieved by crystallization from an aqueous solution of α-CD saturated with N_2_O. The results obtained in the present study showed very good potential for a cheaper production of “cyclodextrin-gas” complexes with a reasonable amount of the captured N_2_O. Therefore, a further extension of their applications is expected, for example, in the preparation of creamy soups and coffees.

## Data Availability

The data presented in this study are available on request from the corresponding author.
